# Plant growth-promoting microorganisms as natural stimulators of nitrogen uptake in citrus

**DOI:** 10.1371/journal.pone.0311400

**Published:** 2025-02-03

**Authors:** Ana Pérez-Piqueres, Belén Martínez-Alcántara, Rodolfo Canet, Raquel del Val, Ana Quiñones

**Affiliations:** 1 Instituto Valenciano de Investigaciones Agrarias, Center for the Development of Sustentable Agriculture, Moncada, Valencia, Spain; 2 IAB, Investigaciones y Aplicaciones Biotecnológicas, S.L., Moncada, Valencia, Spain; Universiti Putra Malaysia (UPM), MALAYSIA

## Abstract

Improving nitrogen uptake efficiency by citrus in Mediterranean areas, where this crop predominates, is crucial for reducing ground-water pollution and enhancing environmental sustainability. This aligns with the *Farm to Fork Strategy (European Green Deal)* objectives, which aim to reduce the use of mineral fertilizers by up to 20% and to eliminate soil contamination from nitrogen entirely. In this context, exploring the potential of plant growth-promoting bacteria application to reduce nutrient inputs is a promising opportunity. The objective of the present study was to evaluate the effect of two *Bacillus subtilis* strains either individually inoculated or in combination with *Saccharomyces cerevisiae* on ^15^N-labeled fertilizer uptake efficiency and physiological parameters. Individual inoculations positively affected tree water potential, leaf chlorophyll concentrations (SPAD-values) and photosynthetic performance, enhancing tree growth. Fertilizer-^15^N use efficiency increased, as did phosphorus and potassium uptakes. Conversely, no response was observed in the trees co-inoculated with *S cerevisiae*. Therefore, PGPB can be considered an interesting means to reduce reliance on synthetic fertilizers in citrus orchards, minimizing the environmental impact and promoting sustainable production practices.

## Introduction

In citrus production, nitrogen (N) is the main nutrient to influence fruit quality [1]. In order to maximize yields for commercial markets, excessive fertilizer rates are frequently applied with concomitant salt buildup, phytotoxic effects on plant growth and ground-water contamination [2]. It is estimated that 40–70% of the total N applied by conventional fertilizers is lost to the environment due to different soil dynamics [3]. On the eastern coast of Spain, where citrus cultivation predominates, nitrate contamination in groundwater exceeds the World Health Organization guidelines of 50 mg L^-1^ [4], which has led to major concerns being voiced since the early 1990s [1]. In this context, it is crucial to find alternatives to optimize and reduce the use of nitrogenous fertilizers.

In the past three decades, agronomy has made many efforts to reduce inorganic fertilization by improving crop management and, proper fertilizer source selection, and by synchronizing crop demand with nutrient supply and developing more effective application methods [3]. In the *Farm to Fork Strategy* report of the European Commission (EC) [5], the EC calls for reducing nutrient losses to the environment by 50% and fertilizer use by at least 20% by 2030. In that the same direction, the integration of new-generation technologies like ecological and molecular approaches now leads to other possibilities. One way of reducing chemical fertilizers is to employ plant biostimulants, which stimulate natural plant nutrition processes without increasing nutrient inputs [6]. Biostimulants aim to improve crop nutrient use efficiency, abiotic stress tolerance and fruit quality parameters, or to increase the availability of constricted nutrients in the rhizosphere. Therefore, supplied in addition to fertilizers, biostimulants may lead to lower nutrient application rates.

Plant growth-promoting bacteria (PGPB), which are considered biostimulants, are a beneficial and heterogeneous group of bacteria that can enhance plant growth, increasing agronomic parameters [7]. The scientific literature describing their mechanisms is substantial [8–14]. PGPB improve nutrient uptake by plants via N fixation, and by increasing nutrient availability, promoting the root surface area or enhancing the host’s beneficial symbiosis [11,15]. PGPB can also facilitate plant growth under stress conditions like, drought and salinity [16–18]. When plants are exposed to stress, they respond by increasing ethylene levels, which lead to cell and plant damage. Many PGPB produce the enzyme ACC deaminase, which destroys 1-aminocyclopropane-1-carboxylate (ACC), a precursor of ethylene, lowering plant ethylene levels and, in turn, favoring plant development [19]. PGPB can also act as phytostimulators. Diverse species alter the root architecture and promote plant development given their ability to synthesize and secrete plant hormones like indole-3-acetic acid, gibberellins, cytokinins and certain volatiles [11,20]. In addition, several PGPB possess biocontrol capacity against plant pathogens through competition for nutrients, niche exclusion, induced systemic resistance and antifungal metabolites production [21], which all imply indirect plant growth stimulation. Thanks to all these mechanisms, using PGPB as inoculants is becoming a sustainable practice, and one that can help to optimize and reduce agricultural inputs.

The effect of PGPB on cereal crops and legumes has been widely studied [12]. However, references about fruit trees, mainly apple trees, are scarce [22]. The few studies available about citrus mostly report PGPB performance on seedlings and nursery plants. Wang et al. [23] found increased plant growth, and N and phosphorus (P) uptakes, by inoculating seeds with *Paenibacillus mucilaginosus* in *Poncirus trifoliata*. In mandarin orange, Thockchom et al. [24], accelerated seed emergence and promoted seedlings growth even 1 year after inoculating a PGP bacterium isolated from mandarin roots. Freitas and Aguilar [25] and Giassi et al. [26] increased plant biomass and shortened the nursery time of citrus rootstocks, “Cleopatra” mandarin, Rangpur lime, Volkamerian lemon, Swingle citrumelo and Sunki mandarin after inoculation with rhizobacteria of different origins. In 6-month-old *Citrus macrophylla* trees, Vives-Peris et al. [27] reduced salt-stress damage *via* the inoculation of *Pseudomonas putida* and *Novosphingobium* sp. isolates. However, the literature about employing PGPB in citrus under stress conditions is still very limited. On the eastern coast of Spain, where rainfall is scarce and water requirements for irrigation directly compete with the increasing demand of industry, tourism or domestic needs, microorganism inoculation might be an interesting option to facilitate plant growth.

As well as PGPB, some rhizosphere and soil yeasts exhibit plant growth-promoting (PGP) effects by inhibiting pathogens, producing phytohormones and solubilizing phosphate [28]. Although yeasts are used mainly for the biocontrol of the fungi involved in the postharvest diseases of plant products, especially fruit, a growing number of examples show their significant potential for enhancing plant development [17,29–31]. Additionally, recent evidence indicates that applying yeast can enhance soil enzyme activity under drought stress conditions by, thereby increasing nutrient content in soil and improving the osmotic condition of roots [17,32].

Mixed inoculants are normally used for many crops grown under field conditions because they promote stronger beneficial effects than single strain inocula. Indeed mostly commercial microbial biostimulants are composed of a consortium of different microorganisms. Microbial consortia may increase strains or species´ ability to cope with continually fluctuating conditions in the rhizosphere of inoculated plants on the one hand, and may have synergistic effects on the survival and persistence of other community members that are less competitive, but desirable, strains on the other hand [33]. Co-inoculation strategies of PGP microorganisms with complementary functional traits and ecology can, thus, boost crop growth promoting effects and improving plant performance [34,35].

For nutrient uptake studies, implementing stable isotope techniques (^15^N, ^44^Ca, ^57^Fe) allows fertilizer supplied nutrients movement to be traced in the plant-water-soil system [2]. However, no assays on labeled fertilizers in combination with PGPB have been carried out in fruit trees.

The purpose of this study was to test the performance of the inoculation of two *Bacillus subtilis* strains on fertilizer-N uptake efficiency (NUE) in young citrus trees. Strains were either individually inoculated or, to enhance their potential growth to promote effects, combined and co-inoculated with a *Saccharomyces cerevisiae* strain. Fertilizer-N movement was traced by the ^15^N isotope dilution technique. The capacity of inocula to improve tree tolerance to water stress was also evaluated.

## Materials and methods

### Experimental conditions, plant material and treatments

This study was conducted at the experimental station of the Valencian Institute of Agricultural Research (IVIA) in Moncada (39° 33´N; 24° 24´W; Valencia, Spain). Twenty-four homogeneous 3-year-old "Fortune" mandarins (*Citrus reticulata* Blanco) grafted onto Carrizo citrange (*Citrus sinensis x Poncirus trifoliate*) rootstock were grown individually in 50 L lysimeter pots containing loamy-sandy soil, sand 55.1%, silt 33.1% and clay 11.8%, pH 8.4, with 0.37% total organic carbon concentration, which is representative of the citrus areas on the eastern Spanish coast. Containers were arranged outdoors on benches under a polycarbonate shelter to avoid rain.

Trees were irrigated by a localized drip irrigation system, with two drips per plant (4 L h^-1^) depending on the monthly water requirements. Irrigation used to be done by a force pump, which distributed the standard nutrient solution from March to October following the seasonal distribution citrus nutrition curve [1,36,37]. N fertilization was carried out employing potassium nitrate and calcium nitrate isotopically labelled with ^15^N, with an average enrichment of 4.7 atom % ^15^N excess. The use of isotope-labelled fertilizers guarantees obtaining complete knowledge of the fate of the N applied with the fertilizer (Table 1). Other macro and micronutrients were supplied according to a half strength Hoagland solution. All nutrients were dissolved in deionized water to avoid isotopic fertilizer-^15^N dilution with water N.

**Table 1:** Monthly distribution of macro and micronutrients per plant supplied in the standard nutrient solution during the assay from march to october.

	Mar	Apr	May	Jun	Jul	Aug	Sep	Oct	Total
N (g)^a^	0.5	1.0	1.5	2.0	2.0	1.5	1.0	0.5	10.0
P_2_O_5_ (g)^b^	0.1	0.2	0.3	0.3	0.3	0.3	0.3	0.2	2.00
K_2_O (g)^c^	0.2	0.2	0.4	0.6	0.8	0.8	0.6	0.4	4.00
MgO (g)^d^	0.3	0.6	0.9	1.2	1.2	0.9	0.6	0.3	6.00
Fe (mg)^e^	5.0	10.0	15.0	20.0	20.0	15.0	10.0	5.0	100.0

^a^Nitrogen supplied as potassium nitrate and calcium nitrate 4.7 atom % ^15^N excess.

^b^Provided as phosphoric acid = 1,113 g P_2_O_5_/1000 ml.

^c^Applied as potassium nitrate (N = 13.5% and K_2_O = 46%).

^d^Supplied as magnesium sulphate (MgO = 16%).

^e^Implemented as multiple chelate (4.5% Fe, 0.5% Zn and 1.0% Mn).

The study consisted of four treatments with six replicates per treatment, one-tree per replicate, which were randomized across the experimental area: i) C, control, which received no biostimulant; ii) BSF1, treatment inoculated with *B*. *subtilis* strain IABBSF1, CECT accession number 8381, isolated from solid urban waste compost, a commercially available formulation at 10^8^ CFU g^-1^ (IAB, SL., Moncada, Spain); iii) BS03, treatment inoculated with *B*. *subtilis* strain IABBS03, CECT accession number 7254, isolated from squash leaves, a commercially available formulation at 10^8^ CFU g^-1^ (IAB, SL., Moncada, Spain); iv) BioF, treatment inoculated with a combination of *B*. *subtilis* strain IABBSF1, *B*. *subtilis* strain IABBS03 and *S*. *cerevisiae* strain IABSC03, CECT accession number 13050, isolated from solid urban waste compost, a commercially available formulation at 10^8^ CFU g^-1^ (IAB, SL., Moncada, Spain). Biostimulants were applied 3 times throughout the growing cycle (May, July and September) at a dose of 5 g tree^-1^ diluted in 0.5 L of nutrient solution. Nutrient solution was uniformly distributed manually in the root zone, followed by short irrigation to facilitate inoculum penetration. Prior to their inoculation, the viability of the inoculants in this solution was checked by a standard dilution-plating procedure. The ability of the *B*. *subtilis* and *S*. *cerevisiae* strains to colonize plant roots was verified using rhizosphere soil samples and roots from each treatment, by employing selective media.

### Physiological status determination

In order to evaluate the effect of treatments on plants’ physiological status, measurements were taken of the SPAD index, chlorophyll fluorescence and the net photosynthetic CO_2_ assimilation on the second spring leaf 2 h after dawn. The SPAD index and chlorophyll fluorescence were determined in July and September after completely applying all the biostimulants. Net photosynthetic CO_2_ was established in July and at dormancy (December). A portable chlorophyll meter (SPAD-502; Konica Minolta Sensing, Inc., Japan) was used to measure the SPAD index, a non-destructive measurement of leaf greenness that correlated positively with chlorophyll content. Chlorophyll fluorescence was recorded to monitor plants´ photosynthetic performance, on 30-minute dark-adapted leaves by a chlorophyll fluorometer OS5-FL (Opti-Sciences, Inc., Tyngsboro, USA), with an excitation source intensity of 0.25 μmol photons m^-2^ s^-1^ on the sample surface to determine the basal fluorescence signal (F_0_). Then a saturating light pulse of 7800 μmol photons m^-2^ s^-1^ was applied for 0.8 seconds to measure maximum fluorescence (Fm). The maximum quantum yield of open photosystem II (PS II) (Fv Fm^-1^) was calculated as (Fm-F_0_)Fm^-1^ according to Maxwell and Johnson [38]. A portable programmable gas analyzer LI 6400-092B (Li-Cor, Inc., Nebraska, USA) was used to measure net photosynthetic CO_2_.

### Plant harvesting, sample preparation and plant analysis

At the end of the plant cycle, trees were destructively harvested during dormancy (December). Young (spring and summer flush leaves and twigs of new shoots) and old (leaves and twigs from previous years, trunk and root systems) organs were separated. To quantify plants´ total dry biomass all the fractions were fresh-weighed and a representative sample of each organ was collected. All the samples were washed in non ionic detergent solution, followed by several rinses in deionized water. Then samples were weighed, frozen in liquid N, freeze-dried and dry-weighed. Plant samples were ground in a water-refrigerated mill IKA M 20 (IKA Labortechnik, Staufen, Germany) and stored at -20°C for further analyses.

Macronutrients [P, K, Mg, calcium (Ca), sodium (Na) and sulfur (S)] and micronutrients [Fe, zinc (Zn), manganese (Mn), boron (B) and copper (Cu)] were measured by simultaneous inductively coupled plasma atomic emission spectrometry (ICAP-AES 6000, Thermo Scientific, Cambridge, UK) [39] after nitric-perchloric digestion. The dried plant material (0.5 g) was digested overnight with 10 ml of HNO_3_ on a digestion block at 120°C. Samples were cooled down to room temperature, and 2.0 ml of 705 ultratrace-metal grade HClO_4_ were added and redigested at 220°C until white fumes were produced. The digest product was diluted to 25 mL with ultrapure water [40] and nutrient concentrations were subsequently measured. The total N concentration and ^15^N abundance determinations were performed with an Elemental Analyzer (NC 2500 Thermo Finnigan, San Jose, USA) coupled to an Isotope Ratio Mass Spectrometer (Delta Plus, Thermo Finnigan, San Jose, USA).

### Calculations

Calculations were done as in Martínez-Alcántara et al., 2012 [1]. Based on the dry weight (DW, g) and the total nutrient concentration data ([Nutrient], %, w/w), the nutrient content for each plant part, was calculated:

Nutrient(g)=[Nutrient]xDW/100

The ^15^N content per plant part was calculated as follows:

N15plantpart(mg)=[Nitrogen]×DW×atom%15Nexcess/10

where atom % ^15^N_excess_ was calculated by subtracting the natural abundance of ^15^N from the atom % ^15^N in each sample. The natural abundance of ^15^N was considered the abundance of atmospheric N_2_, 0.3663 atom %, according to the International Atomic Energy Agency [41]. The N that derived from fertilizer (Ndff) provides accurate information about the amount of N, in all the organs, that derives directly from the applied fertilizer, allowing this N to be discriminated from that coming from soil or plant reserves.


$$N_{dff}(\%)={(}^{15}N_{plant\;compartment}{/}^{15}N_{fertilizer})\times 100$$

### Statistical analysis

Data were subjected to an analysis of variance (ANOVA) to test for significant differences between treatments. Before carrying out any statistical analysis, the normality of all the data was studied by the Kolmogorov-Smirnov test. If the hypothesis of normality was ruled out at the 95% confidence level, data were transformed according to the logarithmic function. Otherwise, data analyses were carried out with the measurements of the variables on their natural scales. The significance of the comparisons made among treatments was analyzed using Fisher´s least significance difference (LSD) test at P < 0.05.

## Results

### Physiological status determination

The effects of treatments on the SPAD index, chlorophyll fluorescence and net photosynthetic CO_2_ assimilation are indicated in Table 2. No significant differences were found in the SPAD-Index in July, when two of the three PGPB inoculations were supplied. The SPAD values in September clearly increased in all the treatments after the three applications of biostimulants. The application of both *B*. *subtilis* strains (BSF1 and BS03 treatments) resulted in significantly higher SPAD values compared to the control trees. On the contrary, no significant changes took place when *Bacillus* combined with *Saccharomyces* was applied. In July, the Fv Fm^-1^ values in the control treatment and treatments BS03 and BioF were lower (0.704, 0.707 and 0.696, respectively) than those obtained with the BSF1 treatment (0.758). In September, the differences in chloropyll fluorescence among treatments became less pronounced. In July, the highest net photosynthetic CO_2_ assimilation, appeared in the trees that received BSF1, with a 36.3% increase in the assimilation rates compared to the control trees. During the dormancy period, when photosynthesis values were low due to poor solar radiation, the net photosynthetic CO_2_ assimilation of all PGPB inoculated trees increased in relation to the control trees (BSF1 by 64.6%, BS03 by 57.0% and BioF by 36.7%).

**Table 2:** Spad Index, chlorophyll fluorescence and net photosynthetic CO_2_ assimilation in young citrus trees.

Treatment^a^	Spad Index		Chlorophyll Fluorescence (FvFm^-1^)		Net photosynthetic CO_2_ assimilation (μmol CO_2_ m^-2^ s^-1^)	
	July	September	July	September	July	December
**C**	51.1 a^b^	70.0 a	0.704 a	0.786 ab	2.51 b	0.79 a
**BSF1**	51.2 a	73.5 b	0.758 b	0.808 b	3.42 c	1.30 c
**BS03**	45.5 a	73.6 b	0.707 a	0.792 ab	2.07 a	1.24 c
**BioF**	43.3 a	72.3 ab	0.696 a	0.777 a	2.40 b	1.08 b

^a^C: Control; BSF1: Inoculation with *B*. *subtilis* strain IABBSF1; BS03: Inoculation with *B*. *subtilis* strain IABBS03; BioF: Inoculation with *B*. *subtilis* strains IABBSF1 and IABBS03 combined with S. cerevisiae.

^b^Values are means (n = 6) of measurements of each treatment. Within each column, values followed by the same letter are not significantly different according to LSD Multiple Range Test at the 0.05 level of probability.

### Growth-promoting effect of the bacterial application

**Fig 1:**
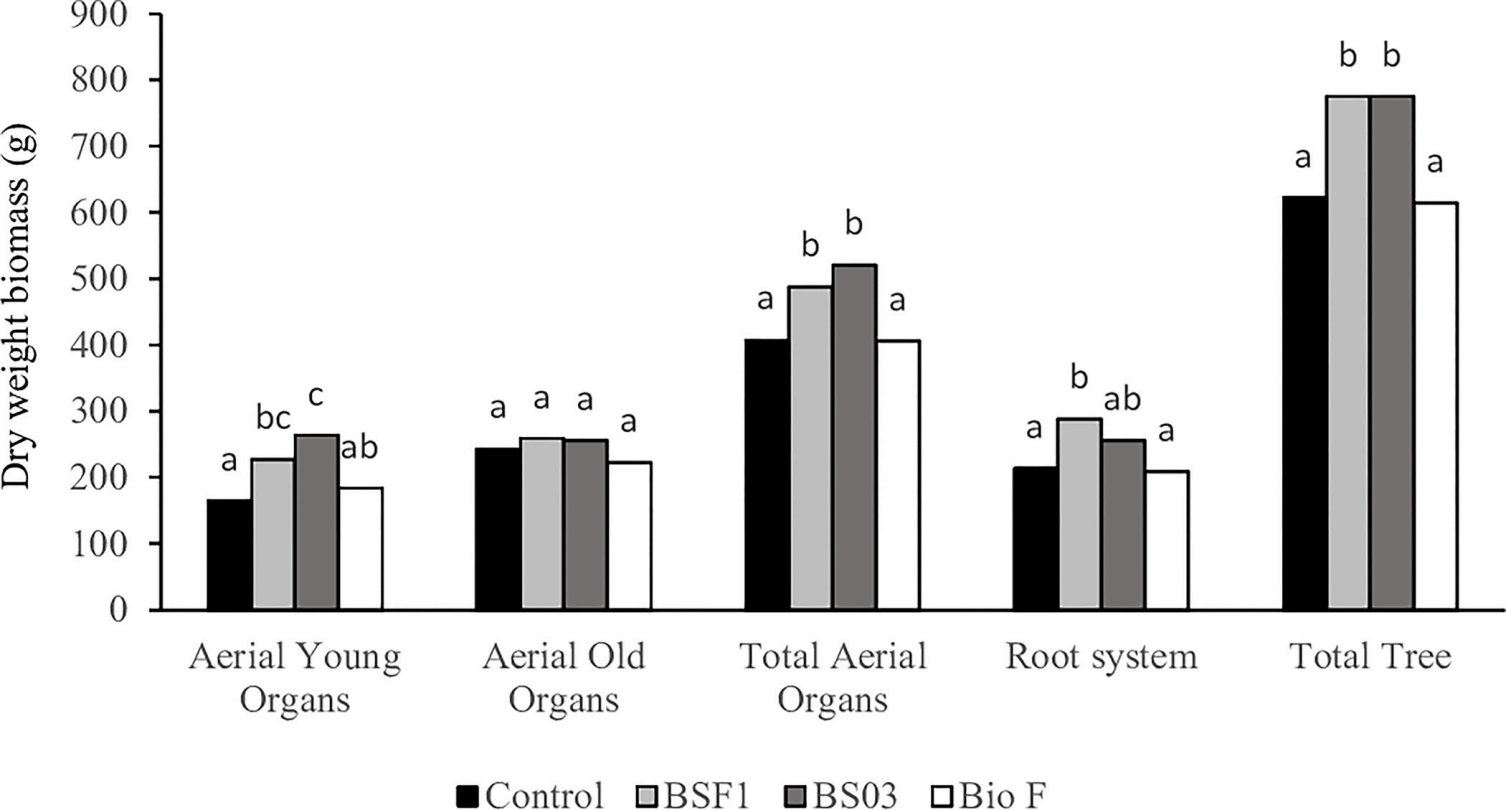
Distribution of dry biomass in the main organs of trees harvested in December (at dormancy). C: Control; BSF1: Inoculation with *B*. *subtilis* strain IABBSF1; BS03: Inoculation with *B*. *subtilis* strain IABBS03; BioF: Inoculation with *B*. *subtilis* strains IABBSF1 and IABBS03 combined with *S*. *cerevisiae*. Values are means (n = 6) of measurements of each treatment. Values followed by the same letter are not significantly different according to LSD Multiple Range Test at the 0.05 level of probability.

Fig 1 displays the effect of treatments on tree biomass. The inoculation with BSF1 and BS03 significantly increased the total biomass by 25% on average. Both treatments stimulated aerial biomass, particularly young organs (new flush leaves and twigs) They also increased root system development, but this effect was statistically significant only in BSF1. The inoculation of *Bacillus* combined with *Saccharomyces* had no effect on tree biomass.

Foliar N concentration was deficient in all treatments (Table 3) according to the standards for the citrus nutritional status diagnosis established by Quiñones et al. [42]. Inoculations did not affect the N concentration in either the aerial part or the root system, but the N content in the whole tree was significantly higher in BSF1 and BS03 because of the increased biomass.

**Table 3:** Nitrogen concentration in main organs (%, dry weight) and total content in whole tree (mg).

Organ	C^a^	BSF1	BS03	BioF	
Summer flush leaves	1.50	1.58	1.44	1.49	
Spring flush leaves	1.72	1.74	1.64	1.74	
Summer flush twigs	0.971	1.01	1.20	1.10	
Spring flush twigs	0.957	0.965	0.886	1.01	
**Young organs**	**1.41 a** ^b^	**1.42 a**	**1.43 a**	**1.45 a**	
Old leaves	1.64	1.63	1.19	1.66	
Branches	0.803	0.857	1.10	0.707	
Trunk	0.575	0.557	0.567	0.447	
**Old organs**	**0.795 a**	**0.769 a**	**0.816 a**	**0.676a**	
**AERIAL ORGANS**	**1.04 a**	**1.07 a**	**1.13 a**	**1.02a**	
Coarse root	0.845	0.673	0.842	0.657	
Fibrous root	1.24	1.48	1.19	1.71	
**ROOT SYSTEM**	**0.914 a**	**0.897a**	**0.940 a**	**0.794a**	
**TOTAL PLANT**	**0.985 ab**	**1.01 ab**	**1.07 b**	**0.942a**
**TOTAL PLANT (mg)**	**6129 a**	**7776b**	**8297 b**	**5776 a**	

^a^C: Control; BSF1: Inoculation with *B*. *subtilis* strain IABBSF1; BS03: Inoculation with *B*. *subtilis* strain IABBS03; BioF: Inoculation with *B*. *subtilis* strains IABBSF1 and IABBS03 combined with *S*. *cerevisiae*.

^b^Values are means (n = 6) of measurements of each treatment. Weighted average was calculated as the summatory of the products of N concentration by biomass of each plant organ (young leaves and twigs; old leaves, branches and trunk; fine and coarse roots), divided by the total biomass (young organs, old organs, root system or total plant, respectively).Within each row, values followed by the same letter are not significantly different according to LSD Multiple Range Test at the 0.05 level of probability.

**Fig 2:**
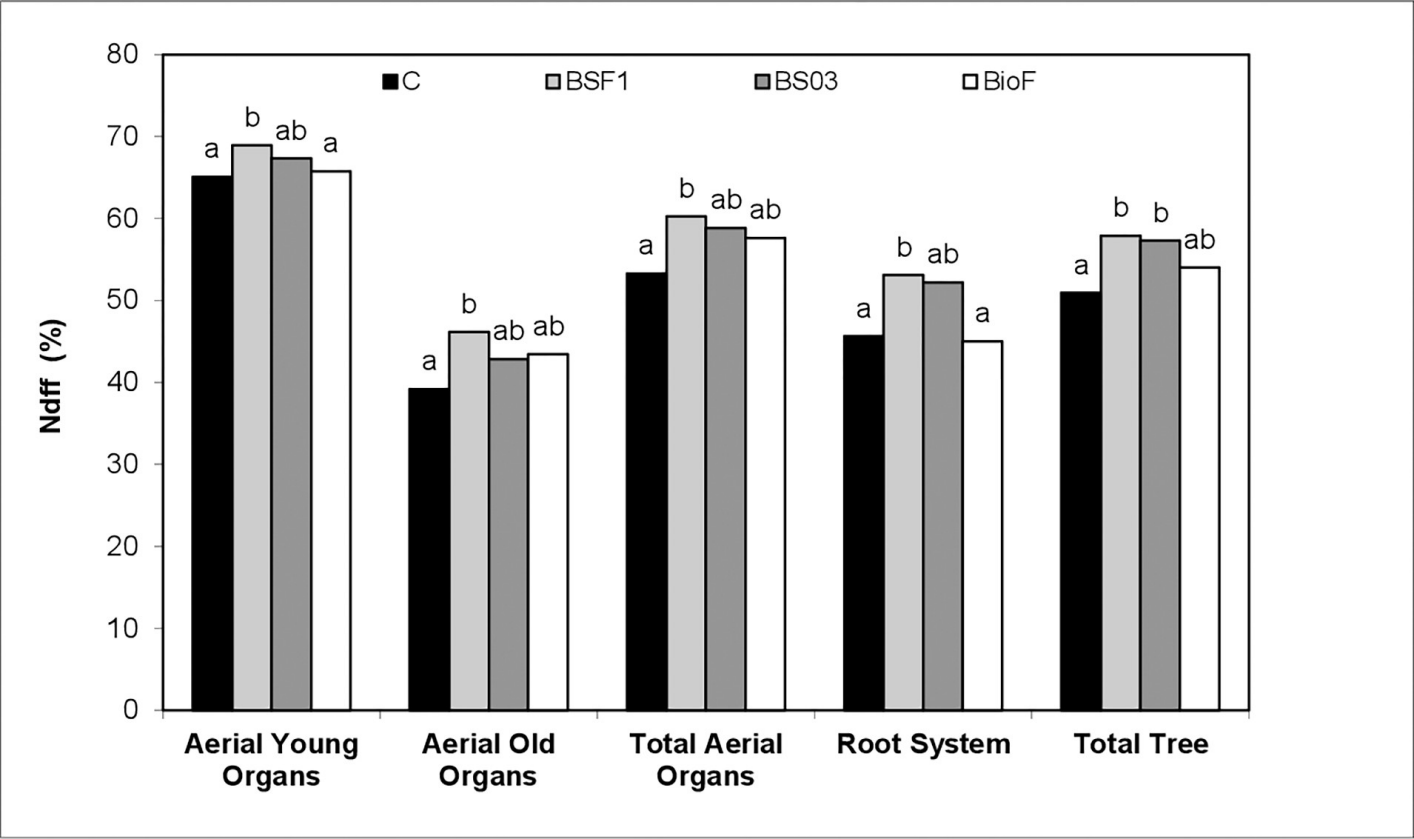
Nitrogen derived from fertilizer (Ndff, %) in the main organs of trees harvested in December (at dormancy). C: Control; BSF1: Inoculation with *B*. *subtilis* strain IABBSF1; BS03: Inoculation with *B*. *subtilis* strain IABBS03; BioF: Inoculation with *B*. *subtilis* strains IABBSF1 and IABBS03 combined with *S*. *cerevisiae*. Values are means (n = 6) of measurements of each treatment. Values followed by the same letter are not significantly different according to LSD Multiple Range Test at the 0.05 level of probability.

Ndff represents the relative contribution of N uptake from the labeled fertilizer to the N built up in each organ. In all plant parts, the highest Nddf was in the BSF1-supplied trees (Fig 2). When considering the average in the whole tree, BSF1 and BS03 obtained the highest Ndff values. Nevertheless, the combination of both *Bacillus* strains and *S*. *cerevisiae* (BioF treatment) resulted in similar values to those found in the control trees, in aerial young organs and in the root system, which had the lowest Ndff percentages. No significant differences in Ndff were found in old organs and the average of the trees of the BioF-supplied trees.

Treatments BSF1 and BS03 showed the highest P contents per tree (Table 4). Nevertheless, foliar concentrations were below the optimal standards (data not shown), even in BS03 where the average plant concentration was significantly higher. The inoculated trees had higher K contents than the non inoculated control, especially BS03, and values were 2.6-fold higher. BSF1 did not modify the K concentration in any of the analyzed plant parts, but BioF increased the K concentration in aerial parts, and so did BS03 in both aerial parts and the root system. Mg, Ca, Na and S absorptions were enhanced by BS03, as shown by plant contents and the Mg plant concentration. Treatment BSF1 increased Mg and S contents, but BioF had no significant effect on the macro nutrient content in the inoculated trees. At the micronutrients level (Table 5), BSF1 and BS03 increased B content per plant, while BioF reduced B absorption along with Zn. Neither the contents nor the concentrations of Fe, Cu and Mn were affected by treatments, which had similar values to those of the control trees.

**Table 4:** Macronutrients concentration in main organs (ppm, dry weight) and total content in whole tree (mg).

Nutrient	ORGAN	C^a^	BSF1	BS03	BioF
**P**	**Aerial organs**	0.0618 a^b^	0.0722 ab	0.0789 b	0.0661 ab
	**Root system**	0.0600 ab	0.0666 ab	0.0872 b	0.0510 a
	**TOTAL PLANT**	**0.0602 a**	**0.0702 ab**	**0.0823 b**	**0.0610 a**
	**TOTAL PLANT (mg)**	**374 a**	**544 b**	**637 b**	**347 a**
**K**	**Aerial organs**	0.642 a	0.758 a	1.27 c	1.05 b
	**Root system**	0.476 a	0.567 a	1.11 b	0.659 a
	**TOTAL PLANT**	**0.579 a**	**0.688 a**	**1.22 c**	**0.919 b**
	**TOTAL PLANT (mg)**	**3592 a**	**5350 b**	**9470 c**	**5614 b**
**Mg**	**Aerial organs**	0.145 a	0.159 a	0.182 a	0.178 a
	**Root system**	0.131 a	0.130 a	0.168 a	0.110 a
	**TOTAL PLANT**	**0.137 a**	**0.148 ab**	**0.179 b**	**0.155 ab**
	**TOTAL PLANT (mg)**	**851 a**	**1148 bc**	**1393 c**	**946 ab**
**Ca**	**Aerial organs**	1.55 a	1.71 a	1.54 a	1.44 a
	**Root system**	2.38 a	1.86 a	2.92 a	1.06 a
	**TOTAL PLANT**	**1.76 a**	**1.78 a**	**2.03 a**	**1.31 a**
	**TOTAL PLANT (mg)**	**10819 ab**	**13794 bc**	**15670 c**	**8132 a**
**Na**	**Aerial organs**	0.0447 a	0.0333 a	0.0515 a	0.0525 a
	**Root system**	0.0127 ab	0.0196 b	0.0187 b	0.00966 a
	**TOTAL PLANT**	**0.0334 a**	**0.0284 a**	**0.0413 a**	**0.0379 a**
	**TOTAL PLANT (mg)**	**206 a**	**219 ab**	**322 b**	**230 ab**
**S**	**Aerial organs**	0.115 a	0.115 a	0.126 a	0.122 a
	**Root system**	0.114 a	0.144 a	0.200 a	0.102 a
	**TOTAL PLANT**	**0.112 a**	**0.126 a**	**0.152 a**	**0.115 a**
	**TOTAL PLANT (mg)**	**697 a**	**979 b**	**1184 b**	**703 a**

^a^C: Control; BSF1: Inoculation with *B*. *subtilis* strain IABBSF1; BS03: Inoculation with *B*. *subtilis* strain IABBS03; BioF: Inoculation with *B*. *subtilis* strains IABBSF1 and IABBS03 combined with *S*. *cerevisiae*.

^b^Values are means (n = 6) of measurements of each treatment. Weighted average was calculated as the summatory of the products of macronutrients concentration by biomass of each plant organ (leaves, twigs, branches and trunk; coarse and fine roots), divided by the total biomass of the plant part (aerial organs, root system, total plant, respectively).Within each row, values followed by the same letter are not significantly different according to LSD Multiple Range Test at the 0.05 level of probability.

**Table 5:** Micronutrients concentration in main organs (ppm dry weight) and total content in whole tree (mg).

Nutrient	ORGAN	C^a^	BSF1	BS03	BioF
**Fe**	**Aerial organs**	44.1 a^b^	46.4 a	41.1 a	37.1 a
	**Root system**	1253 a	1217 a	1545 a	583 a
	**TOTAL PLANT**	**424 a**	**484 a**	**557 a**	**222 a**
	**TOTAL PLANT (mg)**	**260 ab**	**380 b**	**431 b**	**138 a**
**Zn**	**Aerial organs**	18.0 a	16.4 a	16.0 a	12.0 a
	**Root system**	22.2 b	18.2 ab	22.1 b	11.3 a
	**TOTAL PLANT**	**19.2 b**	**17.1 b**	**18.2 b**	**11.8 a**
	**TOTAL PLANT (mg)**	**11.9 b**	**13.1 b**	**14.1 b**	**7.27 a**
**Mn**	**Aerial organs**	18.4 a	13.4 a	16.1 a	14.7 a
	**Root system**	39.7 a	25.4 a	37.8 a	17.0 a
	**TOTAL PLANT**	**24.4 a**	**18.0 a**	**23.6 a**	**15.5 a**
	**TOTAL PLANT (mg)**	**15.1 ab**	**13.9 ab**	**18.4 b**	**9.54 a**
**Cu**	**Aerial organs**	3.30 b	3.17 ab	2.78 ab	2.33 a
	**Root system**	9.64 a	8.19 a	11.6 a	5.66 a
	**TOTAL PLANT**	**5.25 a**	**5.03 a**	**5.79 a**	**3.47 a**
	**TOTAL PLANT (mg)**	**3.26 ab**	**3.92 ab**	**4.51 b**	**2.14 a**
**B**	**Aerial organs**	26.0 a	26.0 a	28.1 a	23.3 a
	**Root system**	11.4 ab	11.8 b	11.9 b	7.83 a
	**TOTAL PLANT**	**20.8 ab**	**20.8 ab**	**22.9 b**	**18.1 a**
	**TOTAL PLANT (mg)**	**12.9 b**	**16.1 c**	**17.7 c**	**11.1 a**

^a^C: Control; BSF1: Inoculation with *B*. *subtilis* strain IABBSF1; BS03: Inoculation with *B*. *subtilis* strain IABBS03; BioF: Inoculation with *B*. *subtilis* strains IABBSF1 and IABBS03 combined with *S*. *cerevisiae*.

^b^Values are means (n = 6) of measurements of each treatment. Weighted average was calculated as the summatory of the products of micronutrients concentration by biomass of each plant organ (leaves, twigs, branches and trunk; coarse and fine roots), divided by the total biomass of the plant part (aerial organs, root system, total plant, respectively). Within each row, values followed by the same letter are not significantly different according to LSD Multiple Range Test at the 0.05 level of probability.

## Discussion

*Bacillus*’ successful ability to colonize the rhizosphere after being introduced into soil and its PGP capacity have been widely reported in different cultures, but have barely been studied in citrus [25,26]. According to our results, the individual inoculation of citrus trees with *B*. *subtilis* had positive effects on their physiological status, growth and nutrient uptake.

The SPAD index, CO_2_ assimilation rates and chlorophyll fluorescence (in strain BSF1) determinations showed that the *Bacillus subtilis* strains inoculation had improved the physiological tree status by the end of the assay. Higher SPAD index values indicated higher leaf chlorophyll contents and, thus, photosynthetic rates, which were supported by the enhanced net photosynthetic CO_2_ assimilation. Several studies have revealed that PGPB-inoculated plants present higher leaf chlorophyll concentrations and better CO_2_ assimilation than non inoculated ones [23,43–45].

In July, low chlorophyll fluorescence values (Fv Fm^-1^) were found in all the trees, clearly below those described in the literature [46,47] for plants growing under appropriate and well-watered conditions, and ranged from 0.75 to 0.85, except for BSF1 inoculated trees. These values revealed plant stress in July, probably due to the high air temperatures usually recorded in the Mediterranean basin in summer months. This ratio is widely employed as an effective tool to quantify stress damage on Photosystem II (PS II) [48] because it reflects the potential quantum efficiency of PS II and is a sensitive indicator of plant photosynthetic performance [38]. Fv Fm^-1^ expresses the probability of an absorbed photon being trapped by the PSII reaction center. Nevertheless, strain BSF1 had a positive effect under stress conditions on the inoculated trees, which showed higher chlorophyll fluorescence values that came close to that of normal range, probably via promoting healthy PSII. These results agree with other authors who have reported higher Fv Fm^-1^ values when using PGPB [48–50] and shown the protective effect of BSF1 on citrus under water stress. In September however, when environmental conditions started to become mild, the Fv Fm^-1^ values record in all treatments fell within the range of the non stressed plants, and no differences were found among treatments.

The individual inoculation with *B*. *subtilis* increased the biomass of young organs and, hence, tree growth. Biomass stimulation by different *B*. *subtilis* strains has been previously reported in herbaceous crops like tomato, okra, African spinach, maize, wheat and barley [51–55], but no studies about fruit trees are available. Certain *B*. *subtilis* strains are capable of biosynthesizing IAA and cytokinins. As these phytohormones are involved in root initiation, cell division and cell enlargement, they enhance lateral and adventitious root development [11,56,57]. Root surface area enhancement means greater nutrient acquisition by plants that, in turn, implies increased plant biomass. In our study, treatment BSF1 showed greater root (biomass) development than the control, which would have produced plant growth stimulation and facilitated tree tolerance to water stress.

Regardless of treatment, all the trees showed deficient foliar N concentrations, probably due to reduced nutrient uptake under stress conditions [58]. Notwithstanding, *Bacillus* inoculation increased N uptake as shown by the N content in treatments BSF1 and BS03, which clearly indicated better NUE from fertilizer. This NUE improvement did not only allow the N concentration to be maintained in trees despite their greater development, but also accumulation in old organs that act as a reserves for subsequent vegetative development [59]. This improvement in fertilizer-NUE was also supported by the Ndff increased values found in both young developing and old organs in the trees supplemented with BSF1, which reinforce the role of fertilizer-N in these organs´ total N pool. A similar effect has been previously reported by Fan et al. [60], who observed enhanced shoot N uptake in tomato after *B*. *amyloliquefaciens* and *B*. *pumilus* inoculation. Kuan et al. [54] reported higher N contents in maize after treatment with *B*. *subtilis*. *Bacillus* can bring about this increase as a result of greater soil N content availability, asymbiotic N_2_ fixation stimulation or more N root absorption. Different studies have shown how PGPB inoculation can increase soil microbial biomass and activity and, in turn, the N that is available for plants. Sood et al. [55] in wheat, Islam et al. [61] in mung bean and Gopalakrishnan et al. [62] in chickpea have demonstrated enhancements in C biomass, dehydrogenase and phosphatase soil activities after plant inoculation with *Serratia* sp., *B*. *cereus* and *Streptomyces* sp., respectively.

Tree P and K contents also increased with *Bacillus* inoculation, especially in treatment BS03, where organ concentrations were significantly enhanced. The capacity of *Bacillus* spp. to solubilize P forms through acidification, chelation or enzymatically is widely reported [12], and is considered one of the most powerful phosphate solubilizers [63] that is used as a biofertilizer under P deficiency conditions. Similarly, *Bacillus* is able to not only release K from insoluble minerals *via* acid production, but to also simulate plants´ ion uptake systems [64]. The marked increase in K content in BS03, whose values were more than 2-fold higher than in the control, was especially interesting because the inoculation of this *Bacillus* strain can improve K fertilization in areas where irrigation water has high Mg levels, a cation that hinders K absorption because of its antagonist effect. These results show the ability of PGPB to enhance NUE, which can be used to reduce chemical fertilization. In wheat, Sood et al. [55] obtained increased plant biomass and yield after applying 80% of the recommended N, a P fertilizer rate in combination with the isolates of *Serratia* sp. and *B*. *subtilis*. Similarly in maize, Kuan et al. [54] reduced by one third the recommended N fertilizer doses by inoculating isolates *B*. *subtilis* and *B*. *pumilis*. In tomato, *B*. *subtilis*, *B*. *atrophaeus* and *B*. *pumilis* promoted plant biomass and height under low N-amended soil conditions [53]. Our results agree with these studies because they indicate the interesting potential of using *B*. *subtilis* in citrus trees to reduce N fertilizers *via* enhancements in fertilizer-NUE.

Many studies have demonstrated that the combined inoculation with beneficial soil microbes can improve crop growth to a much greater extent than individual inoculation [65]. Apart from the present study testing the individual inoculation of two *B*. *subtilis* strains, trees were treated with a combination of both strains and *S*. *cerevisiae*. This yeast can have positive effects on plant development. Gao et al., [66] enhanced plant growth and alleviated the adverse effect of drought after inoculation in rice. Kang et al. [28] observed an increase in gibberellin and organic acid contents in cucumber plants that, in turn, facilitated nutrient uptake and plant growth. Mohamend and Almaroai [67] improved N uptake and grain yield with wheat after the combined inoculation with *S*. *cerevisiae* and *Azotobacter chroococcum*. In our study, however, co-inoculation did not have the positive effects observed on individual *Bacillus* inoculation, such as better plant physiological status (plant biomass, higher SPAD index and chlorophyll contents) and improved NUE. Sometimes, the combination of inoculants does not necessarily have an additive or synergistic effect, but is instead a competitive process [68]. In addition, although several studies have reported that complementarity among different plant growth-promoting traits enhances plant productivity, functionally redundant communities do not enhance plant development [26,69]. Further studies that include the inoculation of *S*. *cerevisiae* alone and with the two *Bacillus* inoculants together in the absence of the yeast, are needed to assist in determining why tripartite inoculation had no effect.

### Conclusions

Improved citrus production applied to meet high-quality fruit demands has very much depended on N fertilizer input. This results in overusing fertilizers and, thus, negatively impacts the environment. The herein presented data support the notion that inoculants (*Bacillus*) can enhance NUE and, thus, help to reduce its buildup in soil that is subsequently prone to be leached to deeper layers. They can also be used to protect citrus trees under water stress by offering a natural and sustainable strategy to address escalating water scarcity. The adoption of a stable isotope technique (^15^N) has enabled the tracing of fertilizer-N movement in the plant-water-soil system. To the best of our knowledge, this study is the first report to use labeled fertilizers in combination with PGPB in fruit trees.

Employing PGPB acts as an eco-friendly management alternative that is particularly interesting for citrus, which is one of the most demanded products on the market for organic produce. However, future studies under field conditions are necessary to confirm the findings obtained in this study.

## Supporting information

S1 Fig Means of dry biomass in the main organs of trees harvested in December (at dormancy). (XLSX)

S2 Fig Means of Nitrogen derived from fertilizer (Ndff, %) in the main organs of trees harvested in December (at dormancy). (XLSX)
